# Mini-Laparotomy Enterolithotomy for Gallstone Ileus in an Elderly Patient: A Case Report

**DOI:** 10.7759/cureus.116656

**Published:** 2026-09-21

**Authors:** Abderrahim Ait Abderrhim, Hicham Assofi, Karim Ibn Majdoub Hassani

**Affiliations:** 1 Visceral Surgery Department, University Hospital Center Hassan II, Fez, MAR

**Keywords:** cholecystoduodenal fistula, enterolithotomy, gallstone ileus, mini-laparotomy, rigler triad, small-bowel obstruction

## Abstract

Gallstone ileus is an uncommon cause of mechanical small-bowel obstruction resulting from migration of a gallstone through a biliary-enteric fistula and occurs predominantly in older adults.

A 70-year-old man with treated hypertension and recurrent, conservatively managed cholecystitis presented with three days of abdominal pain, vomiting, and progressive distension. He was febrile (38°C), tachycardic (110 beats/min), and had a blood pressure of 100/60 mmHg. Laboratory testing showed mild acute kidney injury, mild hypokalemia and hypochloremia, and a moderate inflammatory response. After initial resuscitation, the anesthesiology team classified the patient as American Society of Anesthesiologists physical status IIE, with “E” denoting emergency surgery. Computed tomography demonstrated small-bowel obstruction, pneumobilia, and a 37-mm ectopic gallstone, with a suspected cholecystoduodenal fistula. At surgery, the stone was located approximately 30 cm proximal to the ileocecal valve. Through an open periumbilical mini-laparotomy extending above and below the umbilicus, the viable ileal loop was exteriorized without laparoscopic assistance. A longitudinal antimesenteric enterotomy allowed extraction of a 4-cm stone, followed by transverse closure in two layers without apparent luminal narrowing. No residual gallstones were detected by manual palpation of the gallbladder through the incision; the suspected fistula was not explored. Recovery was uncomplicated, and the patient was discharged on postoperative day four. At one month, the patient was clinically well, but he subsequently missed scheduled appointments and was lost to follow-up. This case illustrates short-term relief of obstruction after mini-laparotomy enterolithotomy alone; long-term biliary outcomes could not be assessed.

## Introduction

Gallstone ileus is an uncommon cause of mechanical small-bowel obstruction resulting from the passage and impaction of a gallstone within the gastrointestinal tract. It develops in approximately 0.3-0.5% of patients with cholelithiasis and predominantly affects older adults, particularly women in their 70s and 80s [1]. It usually develops after formation of a biliary-enteric fistula caused by chronic inflammation of the gallbladder and adjacent bowel. The condition is associated with significant morbidity because of delayed diagnosis, advanced age, comorbidities, dehydration, and the physiological consequences of intestinal obstruction [1-4].

The terminal ileum is the most frequent site of stone impaction because of its relatively narrow lumen. Clinical manifestations are often nonspecific and include abdominal pain, vomiting, abdominal distension, and features of mechanical intestinal obstruction [1,2,5]. Computed tomography (CT) is the preferred imaging modality because it can identify the obstructing stone, the level of obstruction, pneumobilia, and, in some cases, the biliary-enteric fistula. The combination of small-bowel obstruction, pneumobilia, and an ectopic gallstone corresponds to the classical Rigler triad [1,5,6].

Surgical treatment is generally required once mechanical obstruction has developed. However, the optimal operative strategy remains controversial. Options include enterolithotomy alone, enterolithotomy combined with cholecystectomy and fistula closure during the same procedure, or enterolithotomy followed by delayed biliary surgery in selected patients [1,2,4,7-9].

Open mini-laparotomy permits direct bowel exteriorization and enterolithotomy through a limited incision without laparoscopic assistance [10]. Compared with standard laparotomy, the smaller access limits exposure and may require extension if wider exploration is needed. Laparoscopic-assisted approaches provide camera-guided exploration but may be technically challenging in the presence of marked bowel distension [1]. These considerations support individualized selection of the operative approach rather than a claim of superiority.

We report a case of gallstone ileus in a 70-year-old man successfully managed by open mini-laparotomy enterolithotomy alone. The case emphasizes the role of a limited procedure in the setting of acute physiological disturbances and highlights individualized operative decision-making.

## Case presentation

A 70-year-old man was admitted to the emergency department with a three-day history of abdominal pain, recurrent vomiting, and progressive abdominal distension. His medical history included hypertension treated with medication, reported by his family to be well controlled, and recurrent episodes of cholecystitis managed conservatively. Before the acute illness, he was able to walk, wash, and dress independently. No formal frailty assessment was performed during the emergency admission.

On admission, his blood pressure was 100/60 mmHg, heart rate was 110 beats/min, and body temperature was 38°C. Abdominal examination revealed mild distension and generalized tenderness, more pronounced in the right flank. Percussion demonstrated predominantly central and periumbilical tympany, and auscultation revealed increased bowel sounds. No abdominal scars or hernias were identified.

The admission laboratory findings are summarized in Table 1. They showed mild acute kidney injury with elevated urea, mild hypokalemia and hypochloremia, and a moderate inflammatory response. Serum sodium and lactate were normal, and the liver biochemical profile was within reference limits. Fluid resuscitation and correction of electrolyte abnormalities resulted in clinical and laboratory improvement. After initial resuscitation, the anesthesiology team classified the patient as American Society of Anesthesiologists (ASA) physical status IIE, with the suffix “E” denoting emergency surgery [11].

**Table 1:** Admission laboratory findings Abbreviations: HCO3-, bicarbonate; CRP, C-reactive protein; IU, international units. Reference intervals may vary between laboratories.

Laboratory parameter	Patient value	Reference value
Serum creatinine	132 µmol/L	60-110 µmol/L
Blood urea	11.8 mmol/L	2.5-7.5 mmol/L
Sodium (Na+)	138 mmol/L	135-145 mmol/L
Potassium (K+)	3.4 mmol/L	3.5-5.0 mmol/L
Chloride (Cl-)	96 mmol/L	98-107 mmol/L
Bicarbonate (HCO3-)	28 mmol/L	22-26 mmol/L
Lactate	1.2 mmol/L	<2.0 mmol/L
Hematocrit	45%	40-50%
Leukocyte count	11.5 G/L	4.0-10.0 G/L
C-reactive protein	38 mg/L	<5 mg/L
Total bilirubin	11 µmol/L	<17 µmol/L
Alkaline phosphatase	72 IU/L	40-130 IU/L
Gamma-glutamyl transferase	24 IU/L	<50 IU/L
Alanine aminotransferase	22 IU/L	<45 IU/L
Aspartate aminotransferase	19 IU/L	<35 IU/L

An abdominal CT scan demonstrated dilated small-bowel loops consistent with mechanical obstruction, pneumobilia, and an ectopic intraluminal calculus measuring approximately 37.1 mm. These findings supported the diagnosis of gallstone ileus. A cholecystoduodenal fistula was suspected radiologically as the route of stone migration but was not surgically confirmed (Figure 1).

**Figure 1:**
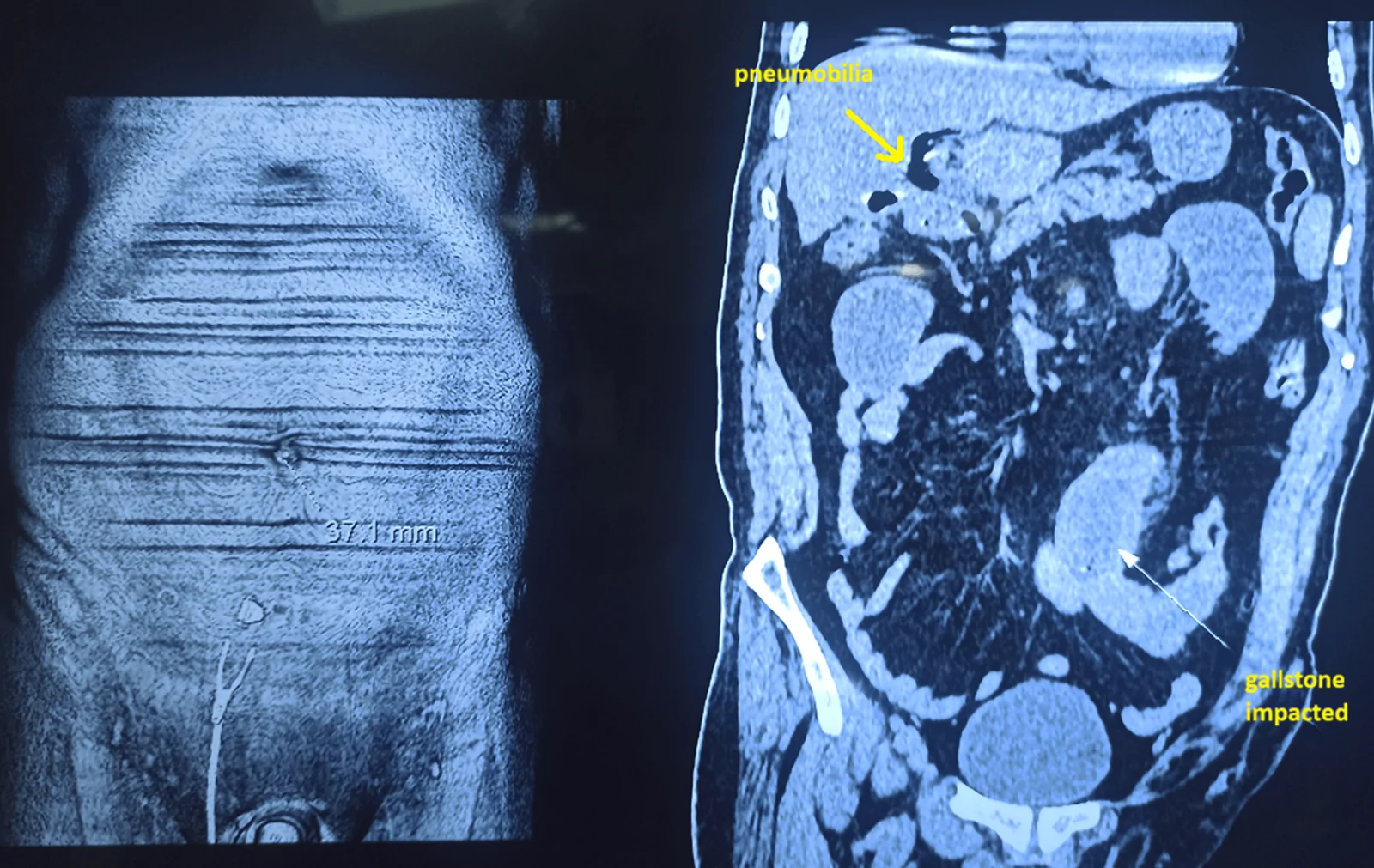
Preoperative Computed Tomography Findings Consistent With Gallstone Ileus The images demonstrate dilated small-bowel loops, pneumobilia, and an ectopic intraluminal gallstone measuring approximately 37.1 mm. The existing annotations identify pneumobilia and the impacted gallstone.

Following initial resuscitation and correction of the preoperative electrolyte and renal abnormalities, emergency surgery was performed. An open periumbilical mini-laparotomy was performed through an incision extending above and below the umbilicus, without laparoscopic assistance. Exploration demonstrated markedly dilated small-bowel loops proximal to an impacted calculus in the distal ileum, approximately 30 cm proximal to the ileocecal valve. The affected bowel segment appeared viable, with no macroscopic evidence of irreversible ischemia or perforation (Figure 2).

**Figure 2:**
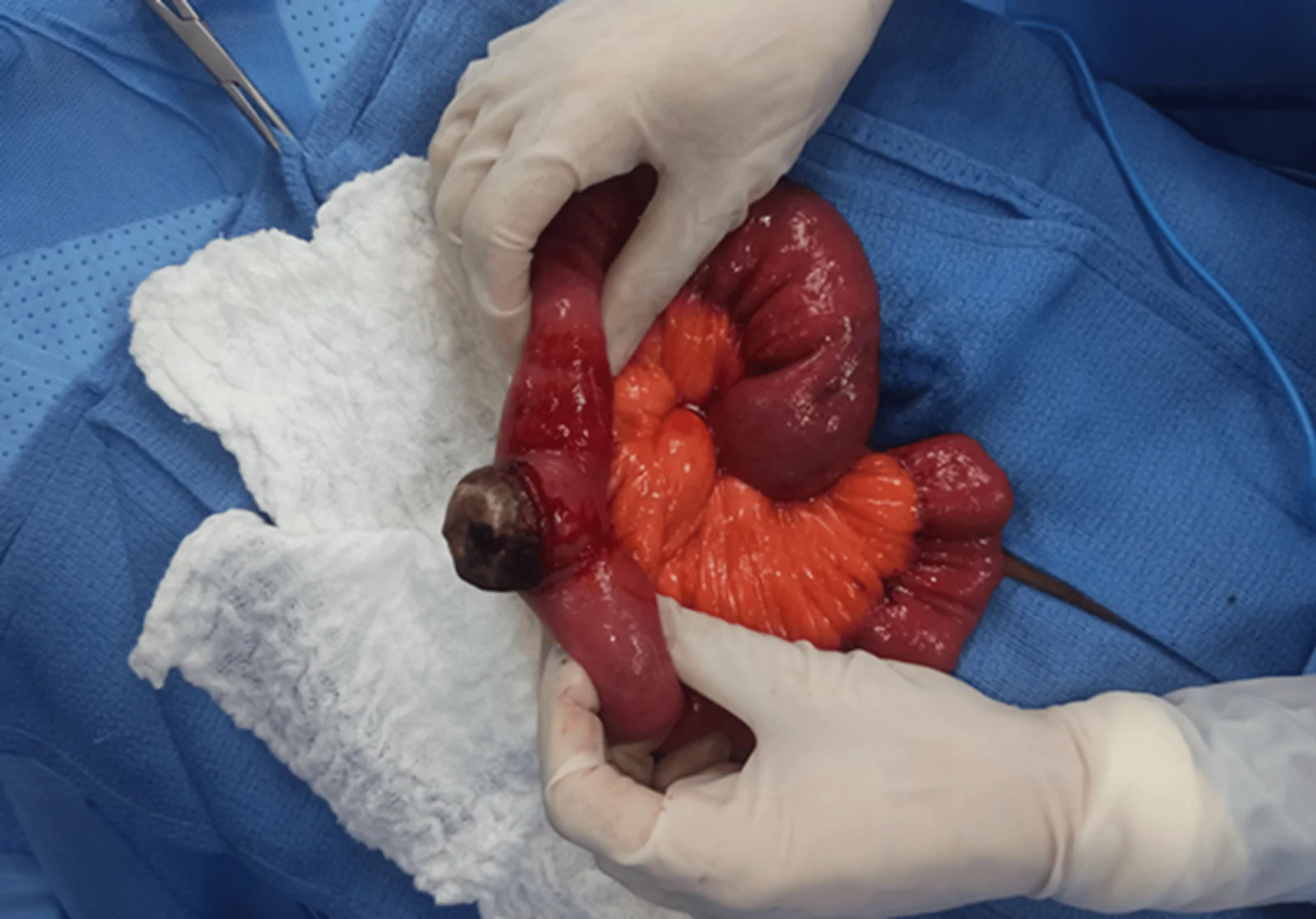
Intraoperative Finding of Gallstone Ileus A large calculus is impacted in the distal ileum approximately 30 cm proximal to the ileocecal valve, with proximal small-bowel dilatation. Intraoperative assessment showed viable bowel without macroscopic evidence of irreversible ischemia or perforation.

Only the ileal segment containing the obstructing gallstone was exteriorized through the mini-laparotomy incision. A longitudinal enterotomy was performed on the antimesenteric border directly over the impacted calculus. An approximately 4-cm gallstone was extracted (Figure 3A). The intestinal wall remained viable following extraction. The enterotomy was subsequently closed transversely in two layers, with no apparent luminal narrowing on intraoperative assessment after closure (Figure 3B).

**Figure 3:**
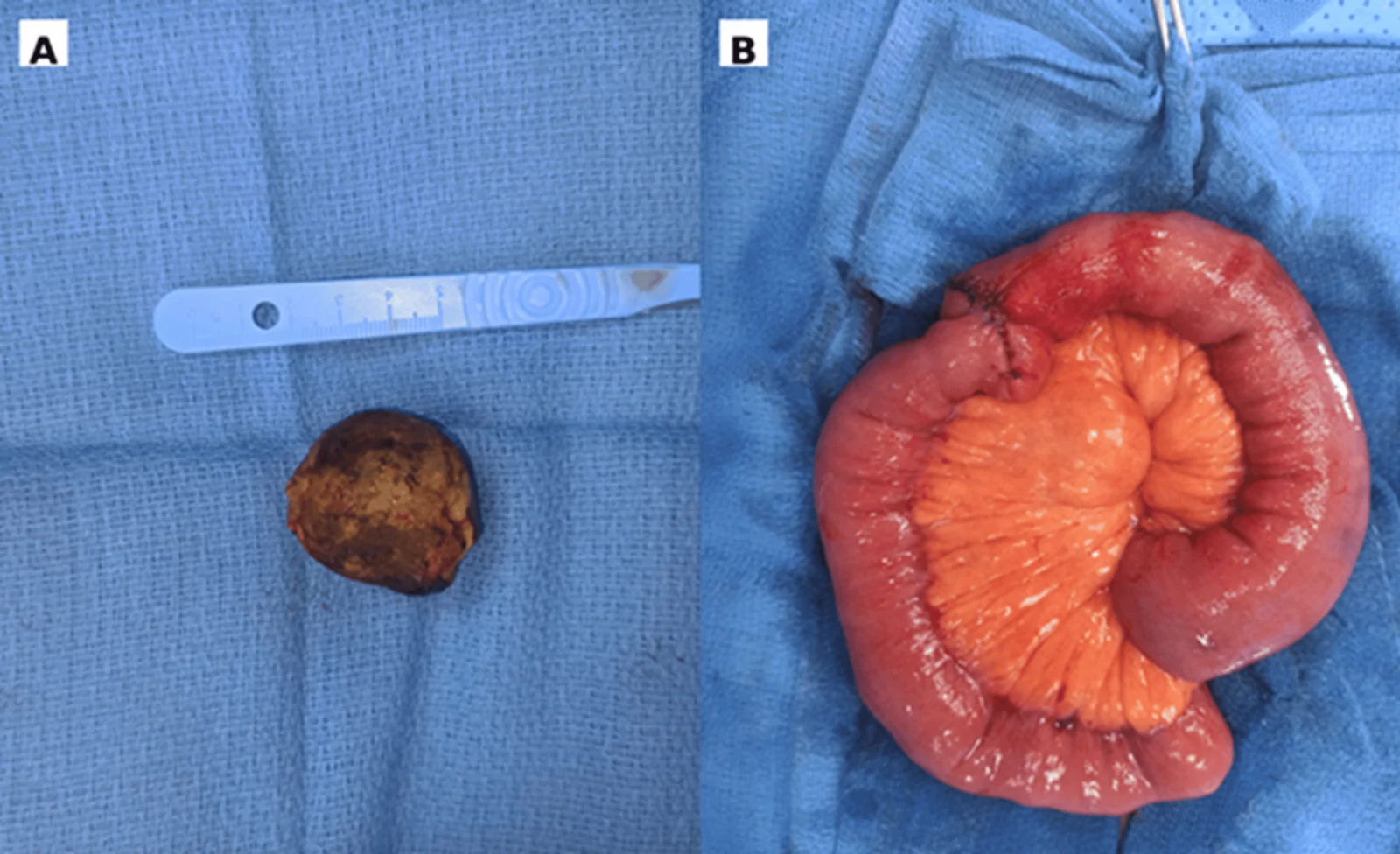
Surgical Management of Gallstone Ileus (A) Extracted gallstone measuring approximately 4 cm. (B) Ileal loop after stone extraction and transverse closure of the enterotomy in two layers. No apparent luminal narrowing was noted on intraoperative assessment after closure.

The gallbladder was assessed intraoperatively by manual palpation through the periumbilical mini-laparotomy incision, with retraction of the wound edges. No residual gallstones were palpable, although palpation alone could not definitively exclude their presence. In the context of the acute presentation, the procedure was limited to relief of the intestinal obstruction, and the suspected cholecystoduodenal fistula was not explored.

The total operative time was approximately 90 minutes. No intestinal resection was necessary, and no intraoperative complication was reported.

The postoperative course was uncomplicated. Intestinal function recovered progressively, and oral feeding was resumed. The patient was discharged on postoperative day four.

At clinical review one month after surgery, the patient was well, with no recurrent abdominal pain, vomiting, or abdominal distension. He did not attend subsequent scheduled appointments and was lost to follow-up after this visit. The one-month review therefore represents the last available clinical assessment rather than a planned endpoint for follow-up.

## Discussion

Gallstone ileus remains an uncommon but potentially life-threatening complication of gallstone disease. Although rare in the general population, its relative importance increases among older patients presenting with mechanical small-bowel obstruction [1-4]. Dehydration, delayed presentation, comorbidities, and acute physiological derangements contribute to the morbidity associated with emergency surgery.

The present case had several characteristic features: recurrent cholecystitis and the CT combination of mechanical small-bowel obstruction, pneumobilia, and a large ectopic gallstone. The stone was found in the distal ileum, approximately 30 cm from the ileocecal valve. CT is valuable because the symptoms are often nonspecific and it can demonstrate the level and cause of obstruction and may identify a biliary-enteric fistula [1,5,6]. In this patient, a cholecystoduodenal fistula was suspected radiologically but was not explored or confirmed intraoperatively.

The principal objective of emergency surgery is relief of obstruction. Enterolithotomy alone addresses the immediate mechanical problem while avoiding additional biliary dissection, whereas a one-stage procedure adds cholecystectomy and fistula closure [2,7,8]. Ravikumar and Williams concluded that enterolithotomy alone was appropriate for most patients, with a one-stage procedure reserved for selected low-risk patients [2]. Rodríguez-Sanjuán et al. similarly found no basis for routine one-stage surgery [7]. In a series of 40 patients, Martínez Ramos et al. observed higher mortality with fistula repair but more late biliary complications when the fistula was left untreated [8].

Historically, the review of 1,001 reported cases by Reisner and Cohen described mortality of 11.7% after enterolithotomy alone and 16.9% after a one-stage procedure [2,12]. These heterogeneous, nonrandomized historical data cannot establish a causal advantage of either strategy. They concern the extent of surgery rather than the comparative effectiveness of mini-laparotomy itself.

The decision to limit surgery was based on the acute presentation and operative findings rather than chronological age alone. The patient had treated hypertension and was independently ambulant and able to wash and dress before the acute illness; frailty was not formally assessed. Although the anesthesiology team classified him as ASA IIE after initial resuscitation [11], his admission findings included fever, tachycardia, borderline blood pressure, mild acute kidney injury, electrolyte abnormalities, and an inflammatory response. These systemic findings did not establish a specific biliary source. Operative assessment showed viable bowel without perforation, allowing enterolithotomy without bowel resection. A mini-laparotomy provided access to the affected segment; Galizia et al. previously reported this approach in a case followed for two years [10]. Such case-level evidence does not establish comparative superiority.

The decision not to explore the suspected cholecystoduodenal fistula prioritized relief of the acute intestinal obstruction while limiting additional biliary dissection. No residual gallstones were palpable, but this finding did not definitively exclude residual cholelithiasis. Long-term management remains controversial, and recurrent gallstone ileus is uncommon but recognized [4,13]. A 2024 meta-analysis of 10 studies involving 293 patients reported a favorable risk-benefit profile for combined enterolithotomy and cholecystectomy, while noting longer operative times for combined procedures and higher morbidity in the fistula-repair group [9]. These findings support careful patient selection rather than a uniform operative strategy and do not establish the comparative effectiveness of mini-laparotomy.

The uncomplicated recovery, four-day hospital stay, and satisfactory one-month review document a favorable early outcome in this patient. However, a single case cannot establish superiority over laparoscopic-assisted enterolithotomy, standard laparotomy, or a one-stage procedure. In the systematic review by Mir et al., 62.6% of recurrent cases with a reported recurrence interval occurred within six weeks, although later recurrences were also documented [13]. This proportion describes the timing among recurrent cases, not the risk of recurrence in all operated patients. Continued follow-up beyond one month is therefore important, with its duration and frequency individualized according to clinical and biliary findings. Loss to follow-up in this case prevented assessment of later outcomes.

Limitations

This report has several limitations. The suspected cholecystoduodenal fistula was not surgically explored or confirmed. Gallbladder assessment relied on manual palpation, which could not definitively exclude residual gallstones. No formal frailty assessment was performed, and the reported baseline functional abilities do not substitute for a standardized frailty score. The patient did not attend subsequent scheduled appointments and was lost to follow-up after the one-month review, preventing assessment of delayed biliary complications, persistent fistula, or recurrent gallstone ileus. No postoperative imaging or endoscopic assessment was performed, and serial postoperative laboratory trends were unavailable. Finally, this single case documents early recovery only and cannot establish comparative effectiveness or long-term safety.

## Conclusions

Gallstone ileus should be considered in older patients presenting with mechanical small-bowel obstruction, particularly when there is a history of gallstone disease. CT is central to diagnosis and may demonstrate small-bowel obstruction, pneumobilia, and an ectopic gallstone. In this patient, open mini-laparotomy enterolithotomy alone relieved the obstruction and was followed by an uncomplicated early recovery. Operative strategy and the need for additional biliary surgery should be individualized according to physiological condition, biliary findings, and operative risk. The one-month follow-up does not establish the long-term safety of leaving the suspected fistula untreated or superiority over alternative approaches. Continued follow-up is necessary to identify recurrent obstruction and delayed biliary complications.
